# Critical Analysis of White House Anti-Drug Plan

**DOI:** 10.19080/gjarm.2017.01.555568

**Published:** 2017-04-27

**Authors:** Kenneth Blum, Lyle Fried, Margaret A Madigan, John Giordano, Edward J Modestino, Bruce Steinberg, David Baron, Michael DeLeon, Thomas McLaughlin, Mary Hauser, Rajendra D Badgaiyan

**Affiliations:** 1 Department of Psychiatry & McKnight Brain Institute, University of Florida College of Medicine, USA; 2 Department of Psychiatry and Behavioral Sciences, Keck Medicine University of Southern California, USA; 3 Division of Applied Clinical Research & Education, Dominion Diagnostics, LLC, USA; 4 Geneus Health LLC, USA; 5 Division of Reward Deficiency Syndrome and Addiction Therapy, Nupathways, Inc., USA; 6 Department of Clinical Neurology, Path Foundation NY, USA; 7 Division of Neuroscience-Based Addiction Therapy, The Shores Treatment & Recovery Center, USA; 8 Eötvös Loránd University, Institute of Psychology, Europe; 9 Department of Psychiatry, Wright State University Boonshoft School of Medicine and Dayton VA Medical Center, USA; 10 National Foundation For Holistic Addiction Studies, USA; 11 Department of Psychology, Curry College, USA; 12 Steer Straight Inc., Vinland, NJ USA and Banyan Treatment Center, USA; 13 Center For Psychiatric Medicine, USA

## Editorial

There is no question that America is experiencing a horrific opiate/opioid epidemic whereby thousands of people are unfortunately dying, and the rate of people seeking treatment is at all -time high. One major problem can be linked to the fact that legal prescriptions for powerful opioid analgesics reached 297 million in 2016. One company that manufactures Oxycontin generated $3.1 billion in revenue in 2017. Moreover, that deaths from prescription drug overdoses have been called the “silent epidemic” for many years. Indeed, approximately one American is dying every 17 minutes from accidental prescription drug overdose [1].

According to some clinicians the “*war on drugs*” was not really a *war on drugs* instead it was a war on addicts. For example, during the war on drugs the catastrophic American drug dependent prisoners of 400,000 in 1980 to over 2.4 million in 2017. Some grim statistics [2] included here are that from 1999 to 2015, more than 560,000 of the people in the USA died of drug overdoses (a death toll larger than the entire population of Atlanta). Overdose deaths from heroin increased four -fold from 202 in 2013, and in 2015 killed more people than HIV/AIDS epidemic at its peak in 1995. There was a 3000% increase in the number of individuals seeking opiate/opioid treatment from 2007 t0 2014 [2]

We the people of America are very concerned about these horrific statistics. As clinicians and addiction scientists, the neurobiological underpinnings of all addictive behaviors represented by the concept of Reward Deficiency Syndrome (RDS) is well understood [3]. However, with the importance of the etiology of this epidemic is the need to treat both the cause and the symptoms to prevent both the initiation of opiates and relapse seem parsimonious [4].

While there are some problems associated with the Affordable Care Act (ACA) including increases in premiums and a shrinking offering of insurance carriers, there are positive benefits such as compulsory treatment of preexisting conditions and prohibition of lifetime caps on benefits among others. Most importantly the ACA‘s inclusion of mental health and Substance Use Disorder(SUD) services as one of ten essential health benefits is consistent with expanding not reducing access to needed treatment. Moreover, states with expanded Medicaid Alternative Benefit Plans -a Federal/State partnership -must cover mental health and SUD treatment services and approximately 30% of those who received coverage through Medicaid expansion have a mental illness (i.e. anxiety, schizophrenia, depression and SUD including opioid addiction). It is noteworthy that according to the American Psychiatric Association 20% of the total 68 million Americans, with a diagnosis this past year of mental health and SUD are counted among those Medicaid recipients with mental illnesses. In fact, Dager and Green [5] pointed out that comorbid depression and SUD result in a downward drift in social-economic status.

Although the White House promises to combat the current opiate/opioid epidemic, beltway insiders provide evidence for real concern. The American Health Care Act (AHCA) from the White House (not dead yet), endorsed replacement of ACA despite it having no congressional approval deemed not conservative enough by some members of Congress. If passed the AHCA will be damaging to all those sufferers and families afflicted by the pharmaceutically induced epidemic. The major differences between the ACA and AHCA involve four key areas: Medicaid, tax credits, insurance mandate, and age -based benefits. In reality, according to Congressional Budget Office (CBO), the proposed AHCA plan will result in a federal cut of about 337 billion with about 14 million Americans becoming uninsured next year and about 24 million by 2026. Critical analysis of the AHCA by the CBO report predicts that in the first year premiums will increase from 15–20% but by year 2026 will be reduced by about 10 percent compared to ACA. Moreover, premiums for the elderly will increase very dramatically.

With this stated the real impact of the new AHCA is on subsequent required treatment and prevention services for mental health and SUD. The AHCA as proposed today will significantly remove benefits from the American health care system. The effect will be increasing demands, as mentioned earlier, placed on to the health care system, a 3,000 percent increase, and many people with SUD being jailed instead of being treated.

**Figure 1:**
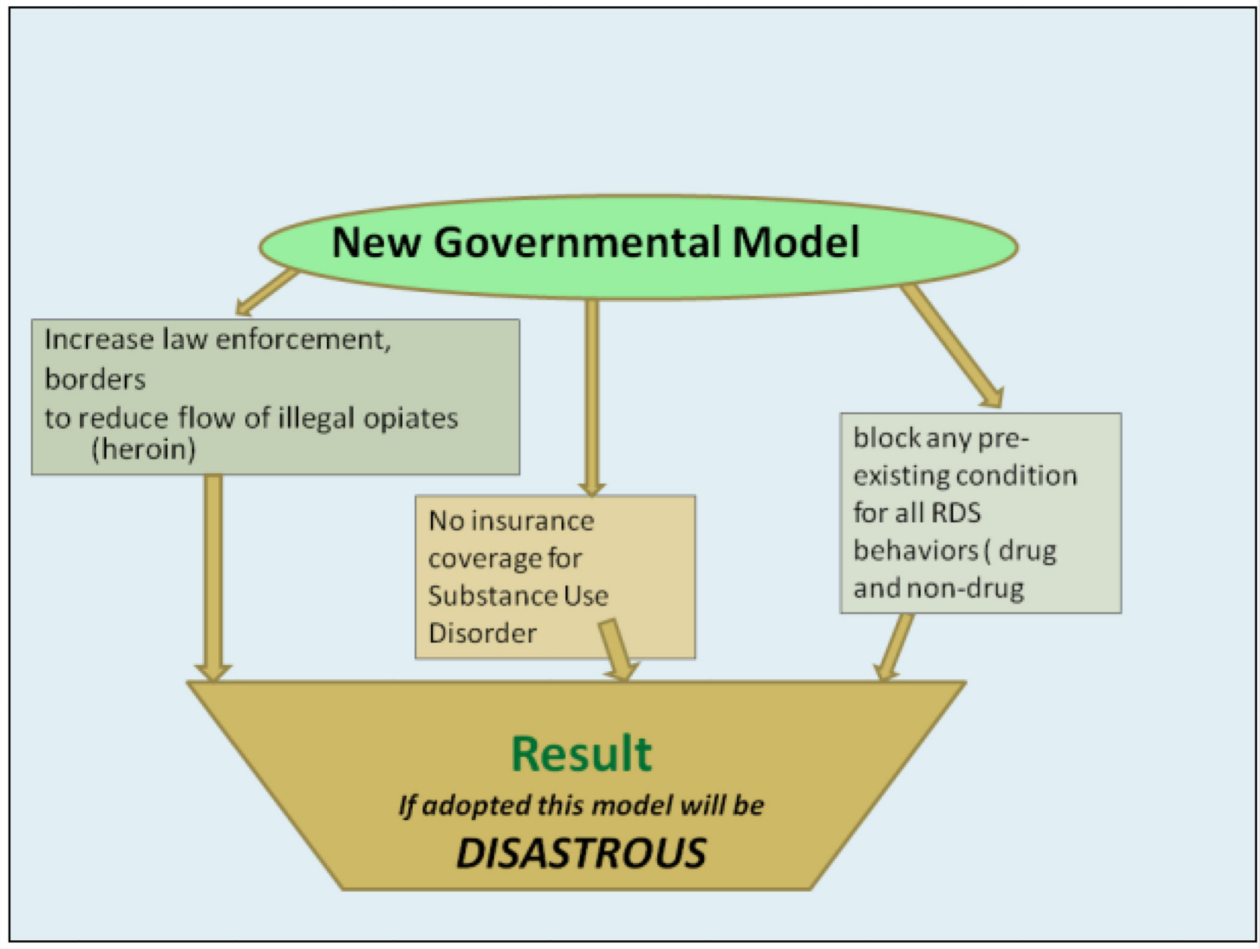
Illustrates the new White House Anti-drug plan.

The White House plan to combat the current opiate/ opioid epidemic involves fighting the supply chain at the borders; reducing treatment options for people who are presently struggling with SUD; locking people into addiction with long-term dispensing of buprenorphine/naloxone combinations and suggesting rehabilitation with no funding allocations [6]. There is no discussion of impacting current laws that allow for the extensive legal prescription of painkillers like Fentanyl and Oxycontin encouraged by pharmaceutical companies (Figure 1).

It is noteworthy that a recent analysis by Puyana et al. [7] revealed that Mexico and United States (US) had indeed, increased border security addressing criminal activities and drug-related trade. This approach of strict law enforcement policies has led to a small reduction of trauma deaths but failed to result in a shift in the drug market to other Central American countries. The effect has been a human crisis with many people suffering injury, and death related to continued drug trafficking despite these efforts “resulting in ***exorbitant loss of lives and cost in productivity across the continent***.” The author’s suggestion of developing health care initiative as an alternative to the actual war approach seems parsimonious.

The adverse effect of reduced insurance coverage benefits for SUD treatment is compounded by the continued long-term expansion of the buprenorphine/naloxone and or methadone treatment. This Opioid Substitution Therapy (OST) approach is rife with issues including anti-reward effects [8]; long-term impact on affect [9]; high addiction liability [10]; genetic antecedents [11]; precipitous withdrawal symptoms [12] and even toxicity especially with methadone [13]. An alternative approach to treating SUD without inducing another addiction involves the concept of pro -dopamine regulation and in particular the evidence -based “*neuroadaptagen*” amino acid enkephalinase inhibitory therapeutic known as KB220 [14–18].

**Figure 2:**
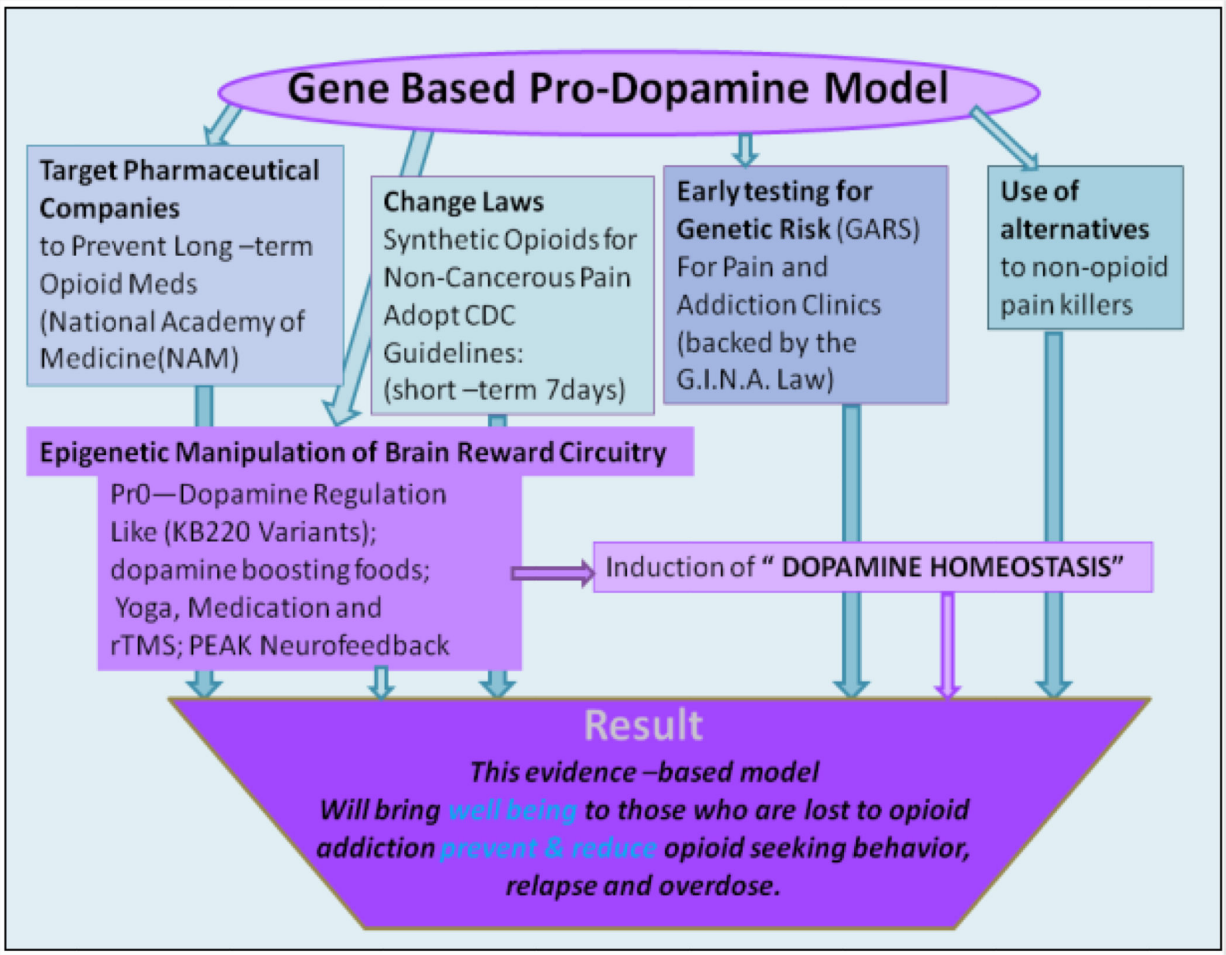
Illustrates a gene based pro dopamine model to help combat the current opiate/opioid epidemic globally.

An alternate approach based on addiction science that could treat this opiate epidemic if embraced by the White House is outlined in Figure 2.

The solution includes:

- Reduction of any long-term use of powerful narcotic opioids by changing existing laws firmly against the widespread legal prescription of opioids like fentanyl and Oxycontin.
- Promotion of expanded treatment for SUD.
- Longer term treatment of from 60–90 days with a lifetime in recovery for those with high genetic severity and risk. We are very concerned that treatment of SUD in America is much too short from only detoxification to 30 days residential.
- Along with genetic risk testing the incorporation of a recently developed RDS scale would help identify patients experiencing abnormal psychological issues [19].
- Induction of pro-dopamine regulation to provide for a more balanced approach leading to “dopamine homeostasis” instead of blocking dopamine function as observed with some FDA Medication Assisted Treatments (MATS) [20].
- Incorporation of some dopamine boosting holistic techniques. Without and induction of epigenetic brain repair “dopamine homeostasis,” any other methodology in our opinion is doomed to fail with high relapse rates.
- Mandatory drug urine screen including a comprehensive analysis of reported drugs [CARD] of patients during treatment and recovery [21] for up to three years to monitor progress and ensure brain recovery [22].

In summary, this critical analysis of the proposed White House plan to combat the current devastating opiate/opioid epidemic in America and globally would be most damaging and could potentially increase the over 52,000 Americans per year who are dying. Despite Secretary Dr. Tom Price’s announcement that the Department of Health and Human Services (HHS) will soon provide $485 million in grants to help states and territories combat opiate/opioid addiction through the Obama Cure Act (OCA, 2016), the concern is that the grants may find their way to nonsensical and unproductive sites. The OCA is an opportunity to combat the iatrogenically induced opioid/opiate epidemic [23]. The current White House Plan, simply stated, is a mindless and damaging false promise and it would be tragic to lose this opportunity.

Finally one of us (MH) attended “Predicting Overdose Death with PDMP Data and Advanced Analytics” on April 16–18 in Atlanta Georgia, where leaders from the government and White House officials discussed the current plan and status of the opiate/opioid epidemic. Dr. Tomas Perez now chairman of the Democratic Party, announced that the party has three main targets 1) childhood obesity; 2) mental health and 3) opioid epidemic, with a laudable goal of securing a special initiative to ensure that the AHCA provides for mandated benefits especially for treatment of SUD, it is unknown whether this mandate will be covered in the actually passing of the AHCA.

## Abbreviations

RDS: Reward Deficiency Syndrome
ACA: Affordable Care Act
SUD: Substance Use Disorder
AHCA: American Health Care Act
CBO: Congressional Budget Office
MATS: Medication Assisted Treatments
OCA: Obama Cure Act
CARD: Comprehensive Analysis of Reported Drugs

## References

[R1] 1https://www.cdc.gov/media/releases/2016/p1216-continuing-opioid-epidemic.html

[R2] 2https://luxuryrehab.co/3000-increase-in-demand-for-opioid

[3] Blum K, Oscar-Berman M, Demetrovics Z, Barh D, Gold MS (2014). Genetic Addiction Risk Score (GARS): molecular neurogenetic evidence for predisposition to Reward Deficiency Syndrome (RDS). Mol Neurobiol.

[4] Blum K, Gold MS, Jacobs W, McCall WV, Febo M (2017). Neurogenetics of acute and chronicopiate/opioid abstinence: treating symptoms and the cause. Front Biosci (Landmark Ed).

[5] Dagher RK, Green KM (2015). Does depression and substance abuse co-morbidity affect socioeconomic status? Evidence from a prospective study of urban African Americans. Psychiatry Res.

[6] Blum K, Gold M, Clark HW, Dushaj K, Badgaiyan RD (2016). Should the United States Government Repeal Restrictions on Buprenorphine/Naloxone Treatment?. Subst Use Misuse.

[7] Puyana JC, Puyana JC, Rubiano AM, Montenegro JH, Estebanez GO (2017). Drugs, Violence and Trauma in México and United States. Med Princ Pract.

[8] Blum K, Chen TJ, Bailey J, Bowirrat A, Femino J (2011). Can the chronic administration of the combination of buprenorphine and naloxone block dopaminergic activity causing anti-reward and relapse potential?. Mol Neurobiol.

[9] Hill E, Han D, Dumouchel P, Dehak N, Quatieri T (2013). Long term Suboxone™ emotional reactivity as measured by automatic detection in speech. PLoS One.

[10] Yarrow A (2017). The billion dollar drug for opioid victims has a problem: it’s Addictive. The fiscal Times.

[11] Blum K, Oscar-Berman M, Jacobs W, McLaughlin T, Gold MS (2014). Buprenorphine Response as a Function of Neurogenetic Polymorphic Antecedents: Can Dopamine Genes Affect Clinical Outcomes in Reward Deficiency Syndrome (RDS)?. J Addict Res Ther.

[12] Blum K, Oscar-Berman M, Femino J, Waite RL, Benya L (2013). Withdrawal from Buprenorphine/Naloxone and Maintenance with a Natural Dopaminergic Agonist: A Cautionary Note. J Addict Res Ther.

[13] Alinejad S, Kazemi T, Zamani N, Hoffman RS, Mehrpour O (2015). A systematic review of the cardiotoxicity of methadone. EXCLI J.

[14] Blum K, Liu Y, Wang W, Wang Y, Zhang Y (2015). rsf MRI effects of KB220Z™ on neural pathways in reward circuitry of abstinent genotyped heroin addicts. Postgrad Med.

[15] McLaughlin T, Febo M, Badgaiyan RD, Barh D, Dushaj K (2016). KB220Z™ a Pro-Dopamine Regulator Associated with the Protracted, Alleviation of Terrifying Lucid Dreams. Can We Infer Neuroplasticity-induced Changes in the Reward Circuit?. J Reward Defic Syndr Addict Sci.

[16] Mclaughlin T, Oscar-Berman M, Simpatico T, Giordano J, Jones S, Barh D (2013). Hypothesizing repetitive paraphilia behavior of a medication refractive Tourette’s syndrome patient having rapid clinical attenuation with KB220Z-nutrigenomic amino-acid therapy (NAAT). J Behav Addict.

[17] Blum K, Febo M, Badgaiyan RD (2016). Fifty Years in the Development of a Glutaminergic-Dopaminergic Optimization Complex (KB220) to Balance Brain Reward Circuitry in Reward Deficiency Syndrome: A Pictorial. Austin Addict Sci.

[18] Febo M, Blum K, Badgaiyan RD, Perez PD, Colon-Perez LM (2017). Enhanced functional connectivity and volume between cognitive and reward centers of naïve rodent brain produced by pro-dopaminergic agent KB220Z. PLoS ONE.

[19] Demetrovics Zsolt, Urbán Róbert, Blum Kenneth (2017). Reward Deficiency Syndrome and Addictive Disorders Psychological Scale.

[20] Blum K, Chen AL, Chen TJ, Braverman ER, Reinking J (2008). Activation instead of blocking mesolimbic dopaminergic reward circuitry is a preferred modality in the long term treatment of reward deficiency syndrome (RDS): a commentary. Theor Biol Med Model.

[21] Blum K, Han D, Femino J, Smith DE, Saunders S (2014). Systematic evaluation of “compliance” to prescribed treatment medications and “abstinence” from psychoactive drug abuse in chemical dependence programs: data from the comprehensive analysis of reported drugs. PLoS One.

[22] Zou F, Wu X, Zhai T, Li Y, Jin X (2015). Abnormal resting-state functional connectivity of the nucleus accumbens in multi-year abstinent heroin addicts. J Neurosci Res.

[23] Blum K, Oscar-Berman M, Dinubile N, Giordano J, Braverman ER (2013). Coupling Genetic Addiction Risk Score (GARS) with Electrotherapy: Fighting Iatrogenic Opioid Dependence. J Addict Res Ther.

